# Stromal Co-Cultivation for Modeling Breast Cancer Dormancy in the Bone Marrow

**DOI:** 10.3390/cancers14143344

**Published:** 2022-07-09

**Authors:** Robert Wieder

**Affiliations:** Rutgers New Jersey Medical School and the Cancer Institute of New Jersey, 185 South Orange Avenue, MSB F671, Newark, NJ 07103, USA; wiederro@njms.rutgers.edu; Tel.: +1-973-972-4871

**Keywords:** dormancy, bone marrow stroma, micrometastases, dormancy models, mesenchymal stem cells, bone marrow fibroblasts, osteoblasts, adipocytes, osteoclasts, endothelial cells

## Abstract

**Simple Summary:**

Cancer cells travel to far away parts of the body before the original cancer can be detected. Breast cancer cells travel mainly to the bone marrow, where they are kept in a quiet, non-dividing, dormant state for many years by cells and proteins in the bone marrow. These metastatic cancer cells wake up at a steady rate over the years and result in incurable disease. This paper outlines how to study the interactions of cells that live in the bone marrow with cancer cells in the laboratory. We describe how to grow mixtures of a variety of bone marrow-residing cells to generate cell layers upon which cancer cells can be grown. Using this system, we can study their effects on cancer cell growth or dormancy and the biology of the interactions. With this approach, we can study how to either eliminate the cancer cells or prevent them from waking up.

**Abstract:**

Cancers metastasize to the bone marrow before primary tumors can be detected. Bone marrow micrometastases are resistant to therapy, and while they are able to remain dormant for decades, they recur steadily and result in incurable metastatic disease. The bone marrow microenvironment maintains the dormancy and chemoresistance of micrometastases through interactions with multiple cell types and through structural and soluble factors. Modeling dormancy in vitro can identify the mechanisms of these interactions. Modeling also identifies mechanisms able to disrupt these interactions or define novel interactions that promote the reawakening of dormant cells. The in vitro modeling of the interactions of cancer cells with various bone marrow elements can generate hypotheses on the mechanisms that control dormancy, treatment resistance and reawakening in vivo. These hypotheses can guide in vivo murine experiments that have high probabilities of succeeding in order to verify in vitro findings while minimizing the use of animals in experiments. This review outlines the existing data on predominant stromal cell types and their use in 2D co-cultures with cancer cells.

## 1. Introduction

Breast cancer (BC) kills more than 43,000 women a year in the United States [1], mostly from metastatic disease. BC cells metastasize to distant organs, including the bone marrow, before patients are diagnosed with local disease [2,3,4,5]. Micrometastases can lie dormant for up to two decades but frequently recur and result in death [6,7,8]. Dormancy is characterized specifically by the capacity of quiescent cells to awaken when factors that initiate dormancy are withdrawn or when the cells are exposed to factors that can trigger an awakening [9]. Understanding the mechanisms governing dormancy and reawakening are important goals for investigators and clinicians.

Studying the factors affecting the behavior of micrometastases in the bone marrow is a complex undertaking in vivo. Animal experiments are expensive, are time consuming and risk failure unless they test viable hypotheses of likely biological scenarios supported by in vitro data. In turn, in vitro experiments must incorporate credible variables found in vivo. There are multiple reciprocal interactions and induced effects between the highly complex spatially organized cellular and structural elements of the bone marrow microenvironments and disseminated cancer cells. The induction of dormancy occurs on multiple levels at the single cell stage, the multicellular stage, and by immune suppression, incorporating a very large number of variables [10]. Modeling the interactions of specific components of the bone marrow microenvironment with individual cancer cells in vitro provides a platform to incorporate these variables found in vivo. This review outlines how the contributions of various stromal elements to cancer cell behavior can be derived from data on their interactions with a variety of malignant and non-malignant cells. These lessons guide the construction of hybrid stromal cultures that have variable and modular components in order to study the cultures’ contributions to co-cultivated cancer cell dormancy and reawakening.

## 2. Interactions of Disseminated Cancer Cells and Bone Marrow Cell Types in the Dormant Niche

Disseminated tumor cells in the bone marrow interact with an extraordinary number of combinations of cell types and with structural and soluble factors. Micrometastases interact with bone marrow cells in various spatial localizations within the marrow, with their subcellular structural segments, through multitudes of receptors and signal pathways, exported molecules, soluble factors, inflammatory cytokines, variable oxygen tension and structural proteins. Structural proteins initiate variable signaling modulated by a variety of chemical modifications, splice variants and variable stiffnesses. Different contexts can initiate opposing effects on disseminated tumor cells (DTCs), resulting in either death, senescence, dormancy or proliferation. It is clear from the complexity of these interactions that there is a necessity for in vitro systems to model them [11].

A number of three-dimensional (3D) and organotypic in vitro models have been developed to study cancer cell dormancy with variable components [12,13,14,15,16,17,18,19,20,21]. Three-dimensional bone-mimicking scaffolds recapitulate rigidity and structural nuances [22,23,24,25,26,27]. Other models, including 3D models, mathematical models, biomaterials, microfluidics and bioreactors, have been developed. These are reviewed expertly elsewhere [15,17,20,21,28] and will not be discussed here. Much of what has been learned from tumor–stromal interactions was derived from 3D models, and those mechanisms will be cited here. However, while 3D models may be more complex and have some advantages over two-dimensional (2D) models, they present a different set of challenges in getting them established and in assessing signaling and molecular interactions with niche elements. The 2D stromal co-cultivation model is relatively less complicated to construct and analyze. Stromal layers can be generated as modular systems with interchangeable and variable niche cells alone or in combination, structural and soluble stromal components, culture conditions and oxygen tensions with the easy introduction of inhibitory antibodies, peptides, small molecules, siRNAs, gene expressions or gene editing vectors. Multimodal analyses using standard molecular biology and imaging techniques provide the necessary versatility to define specific interactions between individual bone marrow elements and cancer cells [28]. Here we outline prior uses of 2D co-cultures to model the dormancy and interactions of cancer cells with stromal components of the bone marrow and propose methods for adding modules to investigate their effects on, and interactions with, cancer cells. Once the interactions are characterized in vitro, they can be validated in specific, focused in vivo hypothesis-driven experiments with high probabilities of succeeding while minimizing the use of animals.

## 3. Components of 2D In Vitro Dormancy Models

### 3.1. Mesenchymal Stem Cells

Mesenchymal stem cells (MSCs) can be obtained and cultured from murine [29,30] or human bone marrow [31]. They encompass a large heterogeneous population of cells that is not well defined [32,33]. MSCs are able to differentiate into several mesenchymal tissues, including bone, cartilage, fat, tendon, muscle and marrow stroma [34]. The minimal criteria that define MSCs were established by the International Society of Cellular Therapy [35]. These include three characteristics: (1) the cells must be plastic-adherent when maintained in standard culture conditions; (2) they must express CD105, CD73 and CD90 and must lack expressions of CD45, CD34, CD14 or CD11b, CD79α or CD19 and HLA-DR surface molecules and (3) the fibroblastoid cells must be able to differentiate into osteoblasts, adipocytes and chondroblasts in vitro [35], which have their own distinct gene expression signatures [36]. The cells also stain positive for nucleostemin, which declines with lineage differentiations [37]. MSCs require forkhead box P1 (FOXP1) to maintain a self-renewal capacity and to prevent senescence. FOXP1 expression declines with age in bone MSCs, a trend inversely correlated with the senescence marker p16^INK4a^, the transcription of which it directly represses [38]. FOXP1 regulates the cell-fate choices of MSCs through interactions with the CEBPβ/δ complex and recombination signal binding protein for immunoglobulin κ J region (RBPjκ), key modulators of adipogenesis and osteogenesis, respectively. As FOXP1 is depleted, it results in an increased bone marrow adiposity, decreased bone mass and impaired MSC self-renewal capacity in mice [38].

MSCs maintain their plasticity [39], stemness and self-renewing capacities in culture medium through the inclusion of L-ascorbic acid 2-phosphate (Asc-2P) and fibroblast growth factor-2 (FGF-2) [36,40]. Asc-2P acts through its role as an antioxidant and inducer of hepatocyte growth factor (HGF) production [40]. FGF-2 promotes the self-renewal and inhibits the senescence of MSCs [41,42,43,44,45], effects mediated though FGF receptor 2 (FGFR2) [46]. The effects are also mediated through the activation of Akt and the inhibition of glycogen synthase kinase-3β (GSK-3β), which is necessary for osteogenic differentiation [47] and adipocyte differentiation [48]. The maintenance of the MSC self-renewal is also mediated through increased levels of beta-catenin, transcriptions of c-myc and cyclin D1 through the activation of the beta-catenin/T-cell factor (TCF) complex [46] and increased levels of Wnt-5a [49]. While Wnt-5a promotes the maintenance of MSCs in the bone marrow, it enhances osteogenesis in cultures [49]. FGF-2 increases both the osteogenic and chondrogenic differentiation potentials of MSCs by inactivating TGFβ and insulin-like growth factor-I (IGF-I) signaling [50] through the induction of SOX2 [51] and of adipose stem cells [52]. TGFβ induces senescence in MSCs through the production of reactive oxygen species (ROS) [53]. FGF-2 also increases preadipocyte early differentiation [54,55] through FGFR1 [56], MEK/ERK-mediated C/EBPα and peroxisome proliferator-activated receptor gamma (PPARγ) activation [57]. Connective tissue growth factor (CTGF) induces MSC fibroblast differentiation [58].

In vivo, endogenous factors, such as PPARγ2, Wnt, IGF-1, growth hormone (GH), FGF-2, estrogen, the gp130 signaling cytokines, vitamin D, glucocorticoids, adipokines (such as adiponectin and leptin, as well as adipose-derived estrogen [59]) and recombinant parathormone [60] regulate the homeostatic maintenance of MSC differentiation toward osteoblasts. Alternately, lower levels of IL-11, GH, IGF-1 and Wnt signaling tilt the balance toward adipogenesis [59]. Signaling by Dexras1 [61], intracellular and extracellular calcium ions [62] and c-Met [40] is also adipogenic.

On the way to adipogenic differentiation, MSCs first differentiate into preadipocytes and then undergo a terminal differentiation into mature adipocytes [63]. Initially, Wnt/beta-catenin signaling suppresses the differentiation and increases the MSC and preadipocyte cell mass, but later, the role of Wnt switches to promoting osteogenesis [63]. TGFβ1 then induces osteogenic differentiation [64] and mediates the suppression of adipogenesis by estradiol through CTGF induction [65]. Interestingly, the transcription factor Ebf-1 suppresses both osteogenesis and adipogenesis in the bone marrow [66].

Hypoxia dramatically increases bone marrow-derived MSC expressions of the hypoxia-inducible factor (HIF) family of proteins and increases both the osteogenic and adipogenic differentiation capacities of MSCs [67]. Hypoxia also enhances adipose-derived MSC (adipose stromal cell, ASC) ERK- and Akt-dependent proliferation, associated with marked increases in the binding of HIF-1α to FGF-2, and associated FGF-2 expression levels [68]. However, FGF-2 regulates the directionality of MSC differentiation by inhibiting adipogenesis and promoting osteoblast differentiation [50]. The inhibition of FGF-2 signaling by a lysyl oxidase propeptide promotes adipogenesis by FGF-2 by inhibiting and down-regulating AKT and ERK1/2 and enhancing the PPARγ and the CCAAT-enhancer binding protein (C/EBP)α, two markers of adipogenesis [69]. A low temperature also promotes adipogenic differentiation of bone marrow MSCs in culture with resulting uncoupled respiration and metabolic adaptations, and translocations of leptin to differentiated adipocyte nuclei [70].

Differentiated MSCs retain their plasticity in vitro and are able to be reverted to pluripotential cells that retain the capacity to differentiate in an alternate direction. Various signaling pathways and mechanisms responsible for the alternate osteoblast/adipocyte cell fate decisions have been expertly reviewed by Hu et al. [71].

Commercially available MSC lines, both human and mouse, can be used, but they have some limitations, such as the ability to differentiate. Nevertheless, they have been used for co-culture studies. A Nestin+ human MSC line can be obtained commercially from Lonza (Basel, Switzerland) [12,13,16]. Murine MSC ST2 cells [72], mesenchymal progenitor cell line C3H10T1/2 [73] and TBR31–2 cells [74] are also available commercially, but their immortalized statuses significantly impair the maintenance of the MSC physiological function, such as bone differentiation [36]. These cells can be cultured in low-glucose DMEM/10% fetal bovine serum (FBS) with antibiotics [12,36].

Most bone marrow stroma-cancer cell co-cultivation experiments have been conducted with primarily fibroblast-differentiated MSCs. However, different stromal cell types can be mixed at various ratios to model stroma and deconvolute the contributions of their individual and joint interactions with cancer cells, adhesion molecules, secreted factors and signaling. MSCs can also be induced to differentiate to different quantifiable extents in different directions to assess the phenotypic and molecular effects on co-incubated cancer cells. The exceptional versatility of such individual and hybrid co-cultivation models can provide an understanding of the specific mechanisms of the stromal effects impacting cancer cell behavior and can generate valid hypotheses that can be confirmed in rationally directed in vivo experiments.

### 3.2. Bone Marrow Stromal Fibroblasts

The most common bone marrow micrometastasis co-culture model systems have a bone marrow MSC fibroblast-predominant cell type. These cells can be obtained from bone marrow aspirates or murine long bone marrow flushed and cultured in several specific medium types that allow stromal MSCs to differentiate into fibroblasts and populate the bottom of a dish. Cultures through several passages can generate monolayer-covered 24-well plates for co-culture experiments, which can be conducted in cancer cell culture medium once the proliferation of stroma is no longer required. Stroma become quiescent at their confluence.

Single cell suspensions of bone marrow hematopoietic progenitors flushed from mouse femurs [75,76] or buffy coats from human bone marrow aspirates obtained from the posterior iliac crests of normal volunteers [16,77,78,79] can be cultured in 25 cm^2^ flasks in Gartner’s Medium [79] or Eagle’s Minimum Essential Medium (alpha modified; αMEM), supplemented with 12.5% fetal calf serum, 12.5% horse serum, 10^−6^ M hydrocortisone, 10^−^^4^ M 2-mercaptoethanol and 1.6 mM glutamine and antibiotics [77]. Alternately, cultures can utilize 20% FBS, 2% penicillin/streptomycin, 0.2% gentamicin and 1 μg/L recombinant human fibroblast growth factor at 1.5 to 3 × 10^6^ cells/cm^2^ at 37 °C and 5% CO_2_ [16]. The medium and non-adherent cells should be demi-depleted every 7 days and replaced with fresh medium until adherent stroma reach approximately 50–75% confluence.

Fibroblasts have organ-specific gene expression patterns, making it important for the model to use bone-marrow-derived fibroblasts [80]. Immortalized bone marrow MSCs have been used by investigators in co-culture experiments when the primary interest has been understanding the effects of bone marrow stromal fibroblasts on cancer cells [12,13,16,72,73,74]. The model has exceptional versatility, and stromal fibroblasts from other organs, including the breast, as well as from primary tumors can be used to generate homogeneous or heterogenous stromal layers to determine their molecular and phenotypic effects on co-cultivated cancer cells. To investigate stromal effects, adding a specific stromal cell type to mixed stromal cultures is sufficient to endow a novel phenotype to co-cultivated cancer cells. Investigators can also target different compartments with toxic vectors, such as a herpes simplex virus thymidine kinase (hsv-TK) vector, used with the prodrug ganciclovir (GCV) or acyclovir, in order to selectively eliminate the effects of stromal construct compartments and demonstrate that they are individually necessary in modulating specific phenotypic or trans effects in co-cultured cancer cells [81].

Stroma can be detached by using a trypsin treatment distributed into multi-well tissue-culture-coated plates at 1–1.5 × 10^5^ cells/cm^2^ and cultured to confluence. Primarily fibroblasts differentiate from MSCs when cultured in these bone marrow media [75,77]. The use of non-bone-marrow-derived fibroblast lines can yield a variety of behaviors in co-cultivated cancer cells, reflecting their organ-specific gene expressions [80]. Both estrogen receptor/progesterone receptor+ (ER/PR+) cells [79] and ER/PR- human BC cell lines, which have been selected to preferentially metastasize to bone [82], are inhibited from proliferating on stromal fibroblast monolayers and form dormant single or oligocellular clones when incubated at clonogenic density. Murine bone marrow stroma can suppress the tumor-initiating content, proliferation, invasion and chemosensitivity of a bone marrow tropic MDA-MB-231 BC cell line [82]. The effects can be achieved by direct contact as well as through transwells by MSC culture-derived exosomes overexpressing miR-23b, which suppress the target gene MARCKS, which in turn encodes a protein that promotes cells cycle and motility [82]. Both the ER- MDA-MB-231 and ER+ T47D BC cell lines can remain dormant in bone marrow stroma co-culture through a gap-junction-mediated transfer of miR-127, −197, −222 and −223, which target C-X-C motif chemokine ligand 12 (CXCL12) [83,84]. Other BC cell lines of various metastatic tropisms have been used in dormancy models [20,85]. The transfer of SDF-1α from bone marrow stroma to BC cells in co-culture can down-regulate the expression of a truncated neurokinin-1 receptor (NK1E-Tr) in MDA-MB-231 cells and induce quiescence when stromal cells outnumber BC cells [86].

In our model, ER/PR+ cells establish a dormant state [79] mediated by dual signaling from FGF-2, which is synthesized and exported by bone marrow fibroblasts [87], and by stromal fibronectin, an integral member of the endosteal microenvironment [88]. Microenvironmental fibronectin suppresses the malignant phenotype [89,90] and helps establish the premetastatic niche through integrin α5β1 [91], but fibronectin produced by cancer cells that have reawakened may play a role in progression [92]. Exogenously presented fibronectin contributes to dormancy signaling through integrin α5β1 [79,93]. Integrin α5β1 expression, which is lost with malignant de-differentiations in these cells, is up-regulated by FGF-2 signaling through transmembrane FGF receptors in a positive feedback loop to maintain cell dormancy [79]. ER- cells are not inhibited by FGF-2 and do not enter dormancy in vitro in a fibronectin-FGF-2 dual signaling model [79] but are inhibited by stromal MSCs by direct or transwell co-cultures through the transfer of miRNAs [82,83,84] or SDF-1α [86].

**Figure 1:**
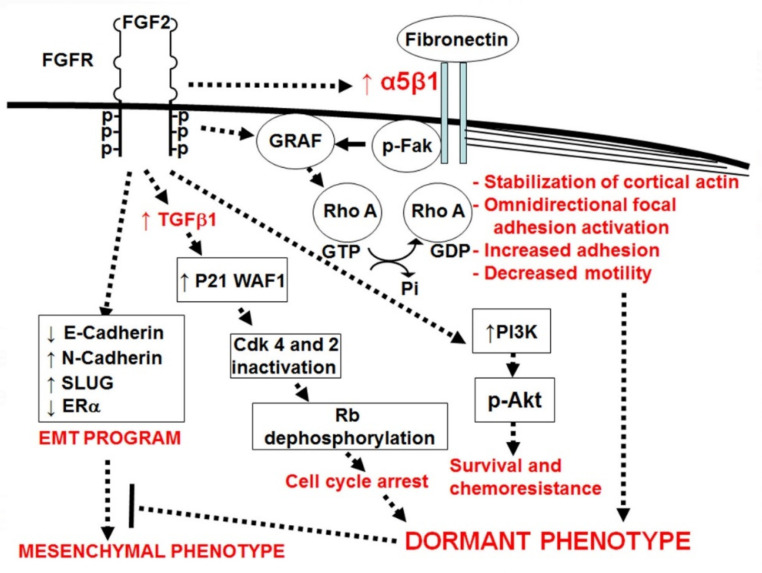
FGF-2 activates FGF receptors, inducing up-regulation of integrin α5β1, which in turn binds stromal fibronectin [79]. FGF-2 induces cell cycle inhibition through increasing TGFβ1 [98], up-regulation of cyclin-dependent kinase inhibitors p21^Waf1/Cip1^, p15^INK4b^ and p27^Kip1^ [97,98], inactivation of Cdk4 and 2 and dephosphorylation of Rb [97]. It induces survival and chemoresistance through activation of PI3K and Akt [79,95]. It induces an epithelial-like dormant phenotype through dual signaling with integrin α5β1 to induce omnidirectional focal adhesion complex activation and increased adhesion [93,95]. FGF-2 and integrin α5β1, in combination, activate the RhoGAP Graf, which is responsible for inactivation of RhoAGTP, causing cortical actin rearrangement and, together with increased adhesion strength, decreased motility [76,93,95]. FGF-2 also activates a mesenchymal program through decreasing E-cadherin and estrogen receptor α (ERα) and up-regulating N-cadherin and SLUG [76]. However, the adhesion signaling suppresses the effects of the mesenchymal program and results in a dormant phenotype [76].

Our lab has developed an in vitro model recapitulating the significant elements of dormancy present in bone marrow stromal monolayers, comprised of a combination of (a) FGF-2- and (b) fibronectin-initiated signaling in (c) ER+ human BC cells (d) incubated at clonogenic density [79] (Figure 1). The latter aspect of the assay is key to ensure that cancer cells interact with only the microenvironment and not each other [20,79]. Cells categorized as dormant after a week of incubation on fibronectin-coated plates in the presence of FGF-2 10 ng/mL [79,94] grow to only a two- to twelve-cell stage, become large, take on an epithelial appearance [79,93], have an increased adherence to the substratum [95] and have a decreased motility [76].

The cells’ appearance, increased adhesion and lack of motility are mediated by an FGF-2-induced re-expression of integrin α5β1, which binds to fibronectin [79], with a consequent omnidirectional focal adhesion complex activation [93]. This results in enlarged, epithelial-appearing flattened cells with increased cytoplasm to nucleus ratios and in a cortical actin rearrangement [79,93]. The actin rearrangement is induced by the RhoGAP Graf-mediated RhoAGTP down-regulation and is dependent on integrin α5β1-mediated signaling [93]. In bone marrow stromal co-cultures, ER+ human cells lie dormant, with FGF-2 provided by the stroma and survival dependent on the stromal fibronectin binding of integrin α5β1 [79].

The non- or hypo-proliferative state of the dormant ER+ BC cells is initiated by FGF-2 [96,97] and is mediated by increased p21^WAF1^ [97,98], p27^Kip1^ and p15^INK4b^ [98] through intracellular TGFβ1 [98], resulting in G1 cyclin complex inhibition and Rb dephosphorylation [97]. FGF-2, on fibronectin-coated plates, also endows the cells with a resistance to cytotoxic therapy mediated through PI3K [79,95]. The role of β1 integrins in providing chemotherapy resistance in cancer has been demonstrated in a number of systems [21]. The process takes place in the setting of ERK pathway activation [96] and constitutive p38 activation by adherence to fibronectin [95].

While FGF-2, in conjunction with fibronectin signaling, induces a dormant phenotype in ER+ human cells, it also induces a mesenchymal gene expression pattern, illustrated by an up-regulation of SLUG and N-cadherin and down-regulation of E-Cadherin and ERα [76]. The ERα down-regulation has been attributed to signaling from stroma, a primary source of FGF-2 in the marrow niche [99]. This in vitro model, demonstrating an epithelial appearance and lack of migration in the setting of an activated mesenchymal program, corresponds to data from DTCs, which are also quiescent, lack the epithelial marker E-cadherin [100] and do not undergo a phenotypic epithelial-mesenchymal transition (EMT) [101]. This supports the current understanding of the microenvironmental control of metastases and provides insights into reconciling the dichotomy of a mesenchymal program masked by an epithelial phenotype in DTCs [100,101].

### 3.3. Osteoblasts

Osteoblast precursors and osteoblasts have reciprocally impactful interactions with cancer cells. Prostate cancer (PC) cells, through extracellular vesicles, enhance the viability of osteoblasts, which, in turn, provide a greater supportive environment for PC cells co-cultivated with them in Boyden chambers [102]. The content of these vesicles is enriched in mRNAs associated with cell surface signaling, cell–cell interactions and translation machinery proteins, which increase the rate of transcription in osteoblasts [102]. BC cells induce Tenascin-W mRNA transcription in bone marrow osteoblasts in vivo, in co-cultures and in transwells through TGFβ signaling [103]. This is mediated through the TGFβ1 receptor Alk5 and a SMAD-4 binding element in the promoter, supporting the migration and proliferation of the cancer cells [103].

However, cancer cells depend on physical interactions with osteogenic cells through gap junctions to increase their intracellular calcium levels [104]. The osteogenic cells serve as a calcium reservoir for cancer cells to promote the formation of micrometastases [104]. Osteoblasts induce proliferation, migration and invasion through the production of hepatocyte growth factor (HGF) in BALB/c-MC cancer cells, mediated by c-Met/HGF receptors [105]. This is a potential mechanism for promoting cancer cell migration from sinusoidal capillaries to the bone marrow space [105]. Osteoblasts also support chemoresistance in cancer cells [106]. MC3T3-E1 pre-osteoblasts and osteoblasts protect ER+ and ER- BC cells from serum deprivation-induced and oxidation-induced cell death by direct contact through integrins and gap junctions [106]. Osteoblasts and MDA-MB-231 co-conditioned medium, with and without heat inactivation, also protect ER- cells but not MCF-7 cells from oxidation-induced cell death, while pre-osteoblast CM alone does not [106]. The induction of osteoblast senescence by p27^Kip1^ promotes metastatic seeding, tumor growth and osteoclastogenesis mediated by IL-6 synthesis in mice [107].

An example of a mutual, sustained effect between osteoblasts and disseminated cancer cells occurs with osteoid-differentiated MC3T3-E1 osteoblasts co-cultured with bone metastatic MDA-MB-231 cells, either in contact or not in contact, sharing a medium [108]. Differentiated osteoblasts induce invasive stimuli in BC cells using MMP13 mRNA and protein, which in turn are induced in the osteoblasts by the cancer cells through soluble factors, including oncostatin M, and the acute-response apolipoprotein serum amyloid A-3 (SAA3) [108]. The invasive effects of MMP13 on cancer cells can be mediated through six novel targets, including the inactivation of chemokine C-C motif ligand 2 (CCL2) and CCL7, activation of platelet-derived growth factor-C (PDGF-C) and cleavage of SAA3, osteoprotegerin (OPG), CutA and antithrombin III [108]. In turn, an MDA-MB-231 co-cultivation in contact with MC3T3 pre-osteoblasts inhibits MC3T3 mineralization in response to ascorbic acid and β-glycerol phosphate, serving as a model of the effects of metastases in the bone marrow [109]

Pre-osteoblasts have a seminal role in harboring the quiescence and preservation of hematopoietic stem cells while differentiated osteoblasts may not [110]. In terms of the former’s interactions with micrometastases, data support a similar relationship of the osteoblast niche with disseminated cancer cells [111,112,113,114,115,116]. Differentiated osteoblasts connect with cancer cells through gap junctions, through which they increase the DTC intracellular calcium and promote colonization [104,117].

Osteoblasts can be isolated from the calvaria of 1–4 day-old neonatal mouse pups by subjecting dissected calvaria to sequential collagenase II or A digestions and culturing the cells in a complete osteoprogenitor medium consisting of Dulbecco’s Modified Eagle Medium (DMEM) and 20% heat-inactivated FBS with antibiotic additives [118,119,120]. Osteoblasts have also been isolated from mincing and collagenase I digesting long bones of adult mice after flushing and discarding bone marrow and from sterile sorting for a lin- cd31- cd51+ sca- cell population, followed by characterizing the cells by using CXCL12(SDF-1)-C-X-C chemokine receptor 4 (CXCR4) immunophenotyping [121]. Alternately, MSCs can be obtained from bone marrow and differentiated in culture into pre-osteoblasts and osteoblasts in αMEM/10% FBS, ascorbic acid-2-phosphate and glycerol-2-phosphate, as noted above [16]. Osteoblast-differentiated MSCs maintain their plasticity along the initial stages of differentiation, with pre-osteoblasts retaining their capability to efficiently convert into adipocytes, and conversely, of adipocytes retaining their capability to convert into pre-osteoblasts [122]. It is therefore important to maintain the cells in a differentiation medium while they populate a dish bottom. Leptin and BMP promote osteoblast osteoid differentiations of bone marrow MSCs in cultures [123], while BMP inhibits late adipogenic differentiation [124]. In hybrid cultures, osteoblasts or bone matrices prevent the osteoblast differentiation of MSCs and ensure a primarily fibroblast differentiation [125]. This serves as an example of the utility of using hybrid cultures with added primary osteoblasts or cell lines to determine their contributions to the behavior of co-cultured cancer cells. The pre-osteoblast cell line MC3T3-E1 can be cultivated in its pre-osteoblast state or differentiated into osteoid-producing cells [36,126] using ascorbic acid [126], ascorbic acid plus dexamethasone and β-glycerol phosphate [36], BMP or leptin [123]. Following their differentiation, cells can be analyzed by using alizarin red staining [36,127], alkaline phosphatase staining [36,126] or a real-time polymerase chain reaction (RT PCR) to detect osteocalcin and RunX-2 gene expressions [128]. Other immortalized osteoblast cell lines, the human osteoblast cell line HS27A and the human fetal osteoblast cell line HFOB have been used in cancer cell co-cultures [129].

**Figure 2:**
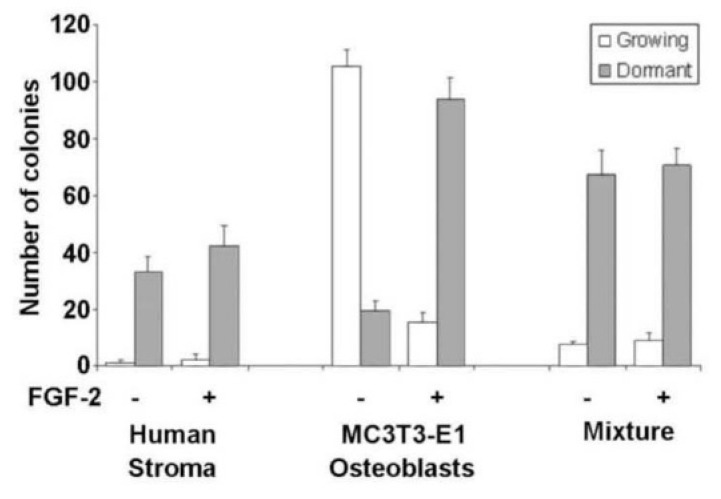
Mixing mouse MC3T3÷E1 osteoblasts with human stroma at a ratio of 1:10 increases the capacity to support dormant MCF÷7 cell clones almost two-fold. FGF÷2 produced by stoma is sufficient to support dormancy [79], with exogenous FGF÷2 having no additional effect on stroma or mixing experiments (*p* > 0.05). However, exogenous FGF÷2 is necessary for dormancy on MC3T3÷E1 monolayers, suggesting they do not produce and export FGF-2. The dormancy support efficiency on MC3T3÷E1 cells is greater than that of stroma by more than two-fold (*p* < 0.05). Error bars ±S.D.

In our model, we combined MC3T3-E1 pre-osteoblasts with human bone marrow stroma fibroblasts and demonstrated the promotion of dormant cell survival by pre-osteoblasts. Our observations of our human HR+ BC line in vitro dormancy model demonstrate that the mouse pre-osteoblast cell line MC3T3-E1 mixed with human stroma at a ratio of 1/10 enhances the support of dormant colonies (Figure 2). The data also show that these pre-osteoblasts alone need exogenous FGF-2 to induce dormancy, while pre-osteoblasts lining the endosteum in vivo express high levels of FGF-2 [130]. It will be important to characterize the expression of FGF and growth factors in planning co-culture models.

### 3.4. Adipocytes

BC cells and adipocytes have reciprocal metabolic effects on each other. Human BC cell lines in transwell inserts can induce an increased lipolysis in co-cultivated adipocytes differentiated from 3T3-L1 cells [131]. In turn, the adipocytes transfer newly produced free fatty acids (FFAs) to the BC cells [131]. The production and transfer of FFAs in adipocytes depend on fatty acid binding protein 4 (FABP4), which combines with FFAs and supports BC cell transmigration. Fatty acids are not transferred to non-transformed MCF-10A mammary epithelial cells, however [131]. Adipocytes contribute to the survival and growth of BC but not of non-transformed cells by increasing the mitochondrial β-oxidation of the transferred fatty acid [131]. This effect is more pronounced in triple-negative BC cells. In human breast tissue, the expression of acyl-coA oxidase 1 (ACOX1) and carnitine palmitoyl transferase 1a (CPT1a), enzymes involved in mitochondrial β-oxidation in BC cells, is higher near adipose breast stroma than in cells near fibrous stroma [131]. Analogously, the expression of FABP4 and hormone-sensitive lipase (HSL) in adipocytes is higher when they are in contact with BC cells [131].

The preadipocyte fibroblast cell lines 3T3-L1 and 3T3-F442A can be obtained commercially and differentiated into adipocytes for use in co-cultivation experiments with cancer cells. The fibroblast lines can be cultured in DMEM/10% FBS to confluence and differentiated into adipocytes 48 h later through the inclusion of 10 μg/mL (1.7 μM) of insulin, 1 μM of dexamethasone, and 500 μM of 3-isobutyl-1-methylxanthine (IBMX) in the culture medium with or without [131,132] indomethacin [16] for 2 days [132] or through the inclusion of 50 nM of insulin alone for 7–14 days [133]. An alternative differentiation medium consists of DMEM/9% horse serum, 250 nM of dexamethasone, 450 μM of IBMX, 1 μM of rosiglitazone and 5 μg/mL (0.9) μM of insulin [36] for 3–14 days. The medium can be subsequently changed back to DMEM/10% FBS after differentiation [133,134].

Bone marrow MSCs can be differentiated in cultures using similar incubation methods with MEM/10% FBS, 1 μM of dexamethasone, 500 μM of IBMX and 10 μg/mL (1.7 μM) of insulin for 14 days [135]. Alternately, human primary adipocytes from reduction mammoplasties, mastectomies or lumpectomies [131,136], mouse mammary tumors [137] or visceral, retroperitoneal, gonadal or subcutaneous white adipose tissue [138] can be used to generate adipose-tissue-derived stromal cells (ASC) that can be differentiated into adipocytes for co-cultures. In one report, adipose tissue was disrupted mechanically, digested with collagenase type I (0.5 mg/mL) and 50 U/mL of dispase and centrifuged at 1000 rpm/mL Floating adipocytes were removed [139], and pelleted ASCs were differentiated into osteoblasts or adipocytes [139]. For the adipocyte differentiation, confluent cell layers were incubated in the adipocyte differentiation medium DMEM/10% FBS and 10 μg/mL (1.7 μM) of insulin, 500 μM of IBMX, 1 μM of dexamethasone and 200 μM of indomethacin for 48–72 h [139], then switched to an adipogenic maintenance medium consisting of DMEM/10% FBS and 10 μg/mL (1.7 μM) of insulin for 24 h. This induction/maintenance cycle was repeated three times, then the cell cultures were switched to a maintenance medium for 10 days, which was changed twice weekly [139].

An adipogenic differentiation can be quantitated by fixing cells with 60% isopropanol, rinsing them with water, staining them with oil red O and extracting them with isopropanol [132]. The optical density at 510 nm is then measured and normalized to counted cell numbers [132]. Alternately, cultured cells can be fixed in 4% paraformaldehyde and stained with oil red O, and the optical density of isoproterenol-eluted lipids can be measured at 490 nm [36]. Differentiation can be detected as well by assaying extracted proteins by using a Western blot and staining them for the adipogenic markers prelipin-1, carnitine palmitoyl transferase 1, hormone sensitive lipase, FABP4 and fatty acid synthase [131]. The culturing of adipocytes from fat tissue can lead to significant gene expression changes that must be considered and carefully controlled when establishing co-cultivation assays [136].

BC cell co-cultures with adipocytes can be established using several approaches. BC cells can be co-cultured with adipocytes in transwell chambers in a high-glucose DMEM/10% FBS with or without Matrigel to determine the effects of adipocyte-produced factors on the biology or behavior of cancer cells, including their proliferation, motility and invasion [134,140], or in mixed 2D cultures [131].

Either ASCs [138] or differentiated ASCs can be used in co-cultivation assays with BC cells [141]. These cells can be mixed with bone marrow stromal fibroblasts at various ratios in 24-well culture dishes, grown to a confluence and then seeded with BC cells at a clonogenic density of 1000 cells per well to simulate the bone marrow microenvironment. The co-cultivation medium should not include differentiation additives for the 7–9 day assays. Alternatively, MSCs can be grown to confluence in 24 well dishes and then differentiated, as above. Then, the medium should be changed to a BC culture medium without differentiation factors, and cancer cells should be added to the mixed cellular monolayer at clonogenic density. The adipogenic differentiation time and completeness can be varied by the number of days, and the extent of adipogenic differentiation should be quantitated in control wells, as above. This will achieve a spectrum of differentiated adipose cells needed to quantitate the differential effects of a partial adipose cell content on the co-cultivated BC cell response. Immunofluorescence antibody studies can be conducted to determine the mutual effects of co-cultured cells on each other.

### 3.5. Osteoclasts

The reawakening of dormant bone marrow micrometastases and the subsequent regrowth of metastatic cancer cells results in the activation of osteoclasts, their maturation and an active bone resorption that enables further cancer cell growth [142]. Most studies on the interactions of osteoclast precursors co-cultivated with cancer cells address the effects of the cancer cell on osteoclast biology [109,143,144,145,146]. However, information on the direct effects of osteoclasts on dormant cancer cells in the bone marrow niche is limited, although the indirect effects on cancer growth through bone erosion and release of growth factors and calcium in the “vicious cycle” in vivo are well known [147]. The establishment of appropriate hybrid stromal co-culture techniques with clonal-density BC cells will enable the study of the effects of osteoclasts on dormancy and reawakening.

Osteoclasts originate in the hematopoietic system from the mononuclear phagocyte lineage, and their maturation entails a fusion of mononuclear cell precursors that express a monocyte/macrophage antigenic immunophenotype [148,149]. Studies have investigated osteoclastic differentiations from a number of cell sources. These include circulating monocytes [148,150], whole bone marrow MSCs [151] and bone marrow macrophages [151], and tumor-associated macrophages from primary and secondary human and mouse mammary carcinomas, which recruit exogenous macrophages [150,152,153]. Other sources of cells able to differentiate into osteoclasts include osteoblasts [154] and commercially available monocyte/macrophage-like cell lines, such as RAW 267.4, an Abelson leukemia virus-transformed cell line derived from BALB/c mice [142], or receptor activator of NFκb (RANK) ligand (L)1- and RANKL2-transfected NIH3T3 cells [155].

Osteoclast differentiation from mononuclear precursors requires macrophage-colony stimulating factor (M-CSF) signaling [150] and RANK/RANKL/OPG, members of the TNF family [142], and TNF receptor-associated factor (TRAF)6, which activates a key transcription factor of osteoclastogenesis, activated T-cell nuclear factor 1 (NFATc1) [151]. RANK, expressed on the surfaces of osteoclast precursors, is activated by binding RANKL, expressed by osteoblasts, mesenchymal cells and cancer cells [142]. OPG, which is a soluble RANKL inhibitor, is also expressed in osteoblasts, bone marrow MSCs and cancer cells, and it moderates osteoclast differentiation and maturation [142]. RANK-induced osteoclast differentiation is mediated through metastasis-associated lung adenocarcinoma transcript 1 (MALAT-1), which in turn suppresses miR-124, a negative regulator of osteoclastogenesis, which decreases the expression of Rab27a, IL-11, NFATc1 and tartrate-resistant acid phosphatase (TRAP) [142]. 1,25-dihydroxyvitamin D3 up-regulates RANKL, CathepsinK, TRAP and MMP-9, genes needed for osteoclast differentiation and function [156]. Cytokines, such as TNF-α, IL-1α and IL-6, and growth factors, such as TGF-β, can induce osteoclast formation from marrow and circulating osteoclast precursors by a mechanism independent of RANKL [150]. Elevated IL-6 levels in adipogenic bone marrow likely activate osteoclast differentiations associated with aging- and obesity-related bone loss [157,158]. Adipogenesis and aging are promoted indirectly by Sirtuin 3, a metabolic regulator of cell senescence driven by mammalian target of rapamycin (mTOR) [158]. Bacterial lipopolysaccharide (LPS) also induces osteoclastogenesis not associated with the RANKL/RANK/OPG axis through the LPS/toll-like receptor 4 (TLR4)/TNFR-2 axis [159]. The process of preosteoclast fusion to form multi-nucleated cells involves the rearrangement of the plasma membrane, mediated by Tks5, a master regulator of invadopodia, inducing fusion downstream of phosphinositide-3- kinase and Src [160]. During this process, differentiated tumor-associated monocytes exhibit an increase in E-cadherin expressions [145].

BC cells induce osteoclast differentiations in MSCs via RANKL, TNFα and other molecules that activate the expression of RANKL in osteoblasts, osteocytes and bone marrow stromal cells [151], but they also induce a decrease in NFATc1 [151]. However, syndecan-1, exported by MCF-7 cells into the conditioned medium, suppresses osteoprotegerin and acts as a positive regulator of osteoclastogenesis [144]. The up-regulation of IL-6 in cancer cells is also mediated through NFκB [161], which in turn can be mediated by CXCL10/CXCR3 [154]. In one study, MDA-MB-231 BC cells induced osteoclastogenesis in human monocytes through M-CSF secretion in a transwell co-culture [143].

As noted earlier, the crosstalk among the variety of cells in the bone marrow microenvironment is reciprocal and affects transitive relationships. The effects by BC-conditioned medium on osteoclastogenesis depend on EGF signaling in BC cells [143] and on mTOR signaling in monocytes [162]. In turn, MSCs co-cultured in transwells with BC cells can induce molecular changes in BC cells, including increased expressions of both RANK and EGFR and a greater capacity to drive the differentiation of peripheral blood monocytes toward osteoclasts. [143].

A variety of approaches have been undertaken to generate osteoclasts for in vitro studies from a variety of cells of hematopoietic origin. Peripheral blood mononuclear cells from either human donors, rats or mice can be isolated by Ficoll centrifugation, followed by red blood cell lysis with 5% acetic acid or Turk’s solution [148,162,163]. Bone marrow mononuclear cells from BM MSCs can be obtained from marrow isolated from the long bones of mice or from human aspirates, which is cultured for 4–7 days and the adherent cells differentiated, as described [109,164]. Bone marrow can be used as well to generate macrophages for osteoclast differentiation [151]. In this approach, freshly obtained bone marrow is placed in a culture in αMEM/10% FBS and M-CSF 50 ng/mL overnight, then non-adherent cells are collected and centrifuged using a Lymphoprep mononuclear cell isolation solution and cultured in an M-CSF-containing medium for 4 days with daily re-feedings [151]. Tumor-associated macrophages can be isolated by using collagenase I digestion, filtering, red cell lysis and culture in M-CSF 25 ng/mL for several days to three weeks in M-CSF, as described [150]. An immortalized monocyte cell line (RW267.4) can be used as well to study osteoclast differentiation and its biology [142]. Alternately, the enforced co-expression of RANKL1 and RANKL2 induces NIH3T3 cells to form multi-nucleated tartrate-resistant acid phosphatase-positive osteoclasts in an in vitro osteoclastogenesis assay system [155].

Osteoclast progenitors from different sources can be cultured in a basal medium of DMEM/10% FBS or αMEM/10% FBS with antibiotics for 4–7 days with day-4 refeedings, the non-adherent cells removed, and adherent cells refed with differentiation medium or incubated in a transwell co-culture with cancer cells [142,162,163,164]. Bone marrow- or cancer-derived macrophages can be maintained in a basal medium with M-CSF until the medium is changed to or added to a differentiation medium.

A differentiation medium can consist of a basal medium with added RANKL (20–100 ng/mL), M-CSF (20-50 ng/mL), PTH (10^−7^ M), TNFα (100 ng/mL), LPS (10 μg/mL) or a BC-conditioned medium, or can consist of various combinations of these factors replenished every 3–4 days for a one to three week period [109,143,150,151,163]. Osteoclast precursor cells can also be cultured in transwells with 0.4 μm pores with BC cells in the inserted upper chamber and the preosteoclasts cultured in the bottom or the wells [143,150,162,163].

The differentiation of osteoclasts can be monitored and quantified by staining for TRAP. The cells are fixed with 10% neutralized formalin phosphate, and TRAP is added in a 0.1 mg/mL acetate buffer containing 0.1 mg/mL of naphthol AS-MX phosphate, a disodium salt substrate for acid phosphatase, 0.6 mg/mL of fast red AL salt (azoic diazo) and 10–50 mM of sodium tartrate [109,143,162,163,164,165]. TRAP-positive cells with ≥3–4 nuclei can be counted [109,143,162,163] or osteoclast areas can be determined by using ImageJ or other comparable software [143]. The quantitation of viable osteoclasts can be determined by measuring their capacity to reduce alamarBlue dye chemically into the growth medium. alamarBlue can be added at a dilution of 1:10 to culture medium for 4 h at 37 °C, and the optical-density 600/570 nm absorbance ratio of the medium can be used to determine the ratio of reduced/fully oxidized alamarBlue [163]. Osteoclast differentiation can also be monitored by determining OPG, RANKL, TRAIL, Cathepsin K, TRAP or MMP-9 mRNA or protein expression [150,156]. For further culture after differentiation, the differentiation medium can be changed back to basic culture medium.

The co-cultivation of cancer cell lines in transwell cultures or the application of a BC cell conditioned medium to osteoclast cultures can promote osteoclastogenesis in monocyte lineage cells [142,143], an effect dependent on EGF signaling in the BC cells [143]. In turn, MSCs co-cultured in transwells with BC cells can induce molecular changes in BC cells, including an increased expression of both RANK and EGFR and a greater capacity to drive the differentiation of peripheral blood monocytes toward osteoclasts [143].

The co-culturing of MDA-MB-231 cells in the same wells in physical contact with bone marrow precursor cells increased their osteoclastogenic differentiation in response to LPS (10 μg/mL) [109]. Head and neck cancer cell lines also induced osteoclastogenesis in macrophage osteoclast precursors from bone marrow [151]. In one study, BM MSCs were mixed in the same well with pre-osteoclast bone marrow mononuclear cells generated from bone marrow by culture in αMEM/10% FBS and 1:10 BC cell 24-h-conditioned medium supplemented with M-CSF for 3 days, cultured to confluence and assayed for osteoclast differentiations by using TRAP assays [164].

These investigations lay out a potential approach to determining the effects of differentiated osteoclasts on BC dormancy in a 2D co-culture system with bone marrow fibroblasts. Osteoclasts can be generated from a variety of sources, including bone marrow, bone marrow macrophages, circulating mononuclear cells, tumor-associated macrophages, osteoblasts, monocytic cell lines and RANKL1- and 2- transfected NIH3T3 cell lines. Once differentiation is confirmed using the variety of available techniques outlined above, differentiated osteoclasts can be dissociated by using trypsin digestion and mixed with trypsin-digested single cell suspensions of bone marrow stroma fibroblasts at various ratios. The mixture of cells can be cultured in 24 well-tissue culture plates in a basal medium consisting of DMEM/10% FBS or αMEM/10% FBS until they are confluent. The osteoclast assay must be used at this point in parallel wells to quantify the number of osteoclasts in the mixed culture, as this number cannot be predicted from the mixing ratios due to the incomplete differentiation of the osteoclast component and to the variable growth rates of the two mixed cellular components. A series of wells with variable osteoclast frequencies should be generated to determine their concentration-dependent progressive effects on BC dormancy. It is advisable that the starting frequency of osteoclasts in a mixed stromal culture be low, probably in the range of 1%, due to their effects on inducing an osteoclastogenic potential in BC cells in co-cultures at higher concentrations [109]. At confluence, the wells can be seeded with BC cells at a clonogenic density of 1000 cells per well and cultured for 7–9 days for BC dormancy or growth determinations. Immunofluorescence labeling can be applied and used in a variety of microscopic imaging or flow cytometry analyses.

### 3.6. Endothelial Cells

#### 3.6.1. The Role of Endothelial Cells in Dormancy

Investigators have demonstrated that the tips of non-dividing vascular endothelial cells in the bone marrow serve as niches for BC dormancy in vivo [12]. Others have determined that endothelial cells induce EMT in cancer cells [166], congruent with the mesenchymal program observed in dormant cells [76]. In addition, endothelial cells in bone marrow vascular niches endow chemoresistance to disseminated cancer cells through integrin-mediated interactions between DTCs and perivascular surface molecules, including the von Willebrand factor and the integrin ligand VCAM1 [18], and by transferring mitochondria to cancer cells through nanotubes [167]. A potential contributor to dormant cancer cell reawakening may be the loss of TSP-1 around sprouting endothelial cells, which accelerate tumor cell outgrowths through TGFβ1 and periostin [12].

The overarching concept governing the study of dormancy in the bone marrow is that all cell interactions modify each other’s molecular and phenotypic behavior. While a bone marrow MSC fibroblast culture induces a dormant, non-motile, non-proliferative mesenchymal phenotype in cancer cells, so do other stromata. One example is the co-cultivation of chorionic villi MSCs with MDA-MB-231 cells [168]. These interactions also significantly reduce the proliferative and migratory capacity of MDA-MB-231 cells, the expression of the BC characteristic cytokines IL-10, IL-12 and CXCL9, and inhibit the tube-forming ability of HUVECs in mixed 3D co-cultures [168].

The role of endothelial cells in dormancy affords a glimpse into the diverse roles of differentiated endothelial cells in both normal somatic and malignant tissues. They have multiple functions besides being perfusing tubes and permeability conductors [169]. Indeed, endothelial cells act as biochemical regulators of cancer cells and the stroma, in constant balance with their tumor [169]. They act as biochemical filters, controllers of hemostasis, biosensors and paracrine regulators, among other functions [169]. Healthy endothelial cells inhibit vascular smooth muscle cell proliferation, monocyte adhesion, thrombosis, cancer cell invasion, inflammation and metastasis [169]. The recognition of the necessity for tumor angiogenesis spawned the realization that tumor cells and endothelial cells have an interdependent relationship, whereby they transfer information between each other [169,170]. Indeed, this has been confirmed in many systems. As an example, the prelecan in healthy endothelial cells inhibits IL-6 in the TME and prevents its tumor-promoting effects. Vascular injury creates a dysfunctional tumor milieu and reverses these inhibitory effects [169].

When investigating the roles of endothelial cells in the dormancy niche using stromal co-cultures and in vivo models, we must consider that direct interactions among cancer micrometastases, endothelial cells and other stromal cells may modify the ability of endothelial cells to support dormancy. This may depend on a list of endothelial cell-, cancer cell- and mesenchymal fibroblast-associated variables, including source, cycle status, differentiation status, passage number, culture conditions and various reciprocal cell ratios, where these interactions may act to modify endothelial cells and induce them to promote cancer cell proliferations. The following examples from the literature underscore the mutual reciprocal effects between cancer cells and endothelial cells.

#### 3.6.2. Cancer Cells Injure Endothelial Cells

Endothelial cells isolated from tumors have unique gene signatures that include a set of 17 genes that are up-regulated compared to angiogenic endothelial cells from normal tissue [171]. These include collagens I and IV, IGF binding protein 7, phosphatidic acid phosphatase 2B, secreted protein acidic and cysteine rich (SPARC), as well as lactate dehydrogenase B (LDHB), among others [171]. In vitro, endothelial cells are injured by incubation with tumor-cell conditioned medium [171]. Connexin 43 in gap junctions, necessary for endothelial cell quiescence, is downregulated by tumors, which allows endothelial cells to respond to angiogenic cues [172]. Tumor-induced angiogenesis is mediated by Fes expressions in cancer cells [173]. Cancer cells also express Ets-1, which induces endothelial cell adhesion and expression and secretion of MMP-9, which endothelial cells recruit to promote angiogenesis and tumor invasion. Ets-1 also promotes endothelial cell-sprouting morphogenesis and decreased endothelial cell chemoattraction and proliferation, resulting in ineffective, leaky capillaries in tumors [174]. Decreased endothelial cell migration induced by cancer cells also contributes to this effect [175]. Co-culture with the human microvascular endothelial cell line-1 (HMEC-1) in 0.4 μm transwell inserts can significantly increase the expression of ANG2 mRNA and protein as well as VEGF protein, and decrease the expression of ANG1 protein in MDA-MB-231 BC cells compared with monocultures [176]. The ratio of ANG1:ANG2 protein, a critical indicator of neovascularization, shifts in favor of ANG2, a phenomenon that correlates with vessel destabilization and sprouting in vivo [176]. In turn, the in vitro angiogenic potential of co-cultivated medium on endothelial cells is markedly increased [176]. Cancer cells with stem cell characteristics induce angiogenesis through HIF1α and VEGF, effects dependent on the retinoic acid pathway and ALDH1A1 [177]. BC cell conditioned medium, or cancer cells in transwell co-cultures up-regulate the adhesion molecules ICAM-1 and VCAM-1 and decrease migration and MT1-MMP and MMP-2 expression in endothelial cells [178]. BC cells or conditioned medium significantly increase hyaluronan synthase-2, hyaluroanan and its receptor CD44 in endothelial cells, and increase the expression and activity of the proteasome β5 subunit [178].

Co-cultivation with either a physical separation or with contact between cancer cells and endothelial cells induces endothelial cell proliferation, migration, and in vitro angiogenesis [179]. Direct-contact co-cultivation induces autocrine growth loops through the up-regulation of matching pairs of angiogenesis-related receptors/ligands coordinately expressed in endothelial cells. These include the TGFβRII/TGFβ3, FGFRII and cysteine-rich fibroblast growth factor receptor (CRF-1)/FGF-7 and FGF-12 chemokine receptor 1 (CCR1), CCR3 and CCR5/regulated on activation, normal T cell expressed and secreted (RANTES; CCL5) and the calcitonin receptor-like gene (CALCRL)/adrenomedullin pairings [179]. Tie-2 receptors are also up-regulated in vitro and in vivo [179]. The totality of the data demonstrate that cancer cell to endothelial cell ratios, the length of time that endothelial cells are in co-culture with cancer cells, proliferative status of the endothelial cells as well as other variables must be considered and controlled when establishing contact co-culture systems so as to increase the probability of consistent data.

#### 3.6.3. Endothelial Cells Modify Cancer Cells

Normal endothelial cells serve a role in resistance to cancer progression. Healthy endothelial cells promote vascular repair and inhibit tumor invasion and metastasis [180]. Factors released from quiescent endothelial cells induce balanced inflammatory signaling, which correlates with decreased proliferation and invasion [180]. Quiescent endothelial cell secretions, mediated through perlecan, inhibit the proliferation and IL-6-mediated invasion of lung and BC cells, and cancer cell pro-tumorigenic and pro-inflammatory signaling in vitro. Endothelial cell-conditioned medium can inhibit the metastatic potential of lung carcinoma cells [181].

However, not all co-cultivation studies report similar conclusions. The context, source, condition of endothelial cells and length of time of exposure to cancer cells during a co-culture experiment affect the response of cancer cells to endothelial cell secretions and co-cultivation. Conditioned medium from a non-contact co-culture with a commercial human umbilical vein endothelial cell line (HUVEC) increases BC cell adhesion, migration and invasion [178]. PECAM-1 expression in endothelial cells promotes tumor cell proliferation in transwell co-cultures and in vivo [182]. These proliferative effects depend on soluble PECAM-1 binding to homophilic ligands, which induces endothelial cells to release TIMP metallopeptidase inhibitor-1 (TIMP-1), leading to tumor cell proliferation [182]. An indirect transwell co-culture of endothelial cell-colony-forming cells (ECFCs), which are late endothelial cell progenitors, and MDA-MB-231 BC cells increases the invasive and migratory phenotypes of both cancer cells and ECFCs. ECFCs have a greater potential for cell division and a capacity for neovascularization than mature endothelial cells [183]. Co-culture induces high levels of secretion of CCL8 through c-Jun by ECFCs, which promotes IL-8 secretion and invasion in MDA-MB-231 cells. In turn, MDA-MB-231 cells enhance MMP-2 secretion and angiogenesis by ECFCs [183].

Endothelial cells derived from an injured vasculature generate a dysfunctional tumor milieu and support tumor progression [169]. Examples of endothelial cells with a dysfunctional state and tumor-stimulating effects are those affected by vascular disease [180]. Endothelial cells rendered dysfunctional by tumor co-culture in a 3D matrix can promote intratumoral pro-inflammatory signaling and spontaneous metastasis, and are able to slow a primary lung tumor growth in vivo [180]. Factors released from dysfunctional endothelial cells activate NFκB and STAT3 in cancer cells, correlating with increased invasion, decreased proliferation and survival in vitro, characteristics of a mesenchymal program [180]. 3D matrix-embedded dysfunctional endothelial cells stimulate intratumoral pro-inflammatory signaling and spontaneous metastasis, while simultaneously slowing a primary tumor growth when they are implanted adjacent to Lewis lung carcinoma tumors [180]. Some experiments can also demonstrate a stimulatory effect by normal endothelial cells on BC colonies when co-cultured for 14 days [184] in a 3D rBM remineralized bone matrix that contains ligands native to an epithelial basement membrane and alginate, an inert biopolymer derived from seaweed [185]. Primary mammary endothelial cells from reduction mammoplasties stimulate the 3D clonogenic potential of normal luminal breast cells and malignant BC cell lines in physical co-cultures and when they are separated in transwell cultures [184]. Due to the long culture conditions, it is likely that during the two-week incubation, cancer cells transfer factors to normal endothelial cells that modify them to a phenotype that promotes tumor cell progression. Under conditions of starvation, co-cultivation with endothelial cells confers, in a contact-dependent manner, a survival advantage, invasiveness, increased mammosphere-forming capacity, an enriched CD44HighCD24Low/- stem cell content and self-renewal ability in BC cells [186]. Co-culture also enhances the metastatic potential of these cells [186]. These effects are mediated by endothelial Jagged1 through the activation of NOTCH [186]. Another study demonstrated that cancer cells secrete ECM1, which, through a feedback loop, induces a NOTCH-mediated endothelial cell promotion of cancer progression by enhancing migration and invasion [187].

PC cell lines demonstrate significantly greater adhesive strength to bone marrow endothelial cells than to HUVECs or lung endothelial cells, an effect dependent on integrin β1 [188]. Bone marrow endothelial cells also increase the invasive potential of PC cells one thousand-fold in a transwell Matrigel colony assay [188]. The differential effect between malignant and benign cell interactions was confirmed in mammary cells. BC cells have markedly greater interaction and elongation when co-cultured with endothelial cells in a 3D matrix than do normal breast epithelial cells [189]. The interaction provides an energetic favorability of cellular deformation, dependent on the cytoskeleton’s ability to elongate and align, prior to breaching endothelial junctions. [189]. Galectin-3 expression in BC cells is required for stabilizing epithelial–endothelial interactions. Co-cultures of epithelial and endothelial cells result in increased galectin-3 secretion in cancer cells and the presence of a proteolytically cleaved galectin-3 in the medium. The proteolytically cleaved galectin-3 displays a much higher affinity for human umbilical vein endothelial cells than does the full-length protein. Increased expression is associated with progression to invasive carcinoma and may lead to an increased invasive potential [190].

#### 3.6.4. Endothelial Cell Sources and Characteristics

Multiple qualities of endothelial cells differentially influence their effects on co-cultivated cancer cells. Their source will affect the outcomes of cancer co-cultivation studies since endothelial cells have organ-specific gene expression patterns [191]. The differentiation status of endothelial cells also matters. Endothelial progenitor cells have an enhanced potential for tumor neovascularization compared with mature endothelial cells [183]. NOTCH-expressing endothelial cells induce cancer progression [187]. As noted above, cancer cell co-cultures in vitro and in tumors in vivo injure endothelial cells [180] and induce NOTCH signaling [187], which in turn promotes cancer progression. Two-dimensional culture causes endothelial progenitor cells to lose early progenitor properties, such as CD34 expression [192]. However, matrix embedding enables endothelial cells to attain a phenotype, secretome and genetic fingerprint similar to endothelial cell progenitors, where they maintain or regain their expression of CD34 [192]. They also re-express leptin, IL-6, IL-8 and monocyte chemoattractant protein-1 and up-regulate TIMP, IL-8, MCP-1, PDGF-BB and angiopoietin-2 [192]. In 2D cultures, the substratum also matters, whereby cells cultured on collagen take on a more in vivo phenotype than cells cultured on tissue culture-coated plates [193]. Endothelial quiescence, in contrast to proliferation and sprouting, also modifies cancer cell proliferative phenotypes [12].

A variety of endothelial sources have been used in cancer co-culture studies, sometimes with conflicting outcomes. The following is a partial list. Endothelial cells have been isolated from human bone marrow [188], cord blood [193,194] and circulating endothelial progenitors [193,195]. Human endothelial colony-forming cells (ECFC) were prepared from the mononuclear fraction of human peripheral blood with CD31-coated magnetic beads [183] and from cord blood using a variety of techniques [193]. Primary endothelial cells have been isolated from normal human breast interstitial stroma and from breast adipose tissue [184,196,197], lungs [182], umbilical veins (HUVECs), [177,198], aortas [192] and placentas [171]. Human dermal microvascular endothelial cells (HDMEC) have been isolated [198] and commercially purchased (HMEC-1) [176]. Primary HUVECs have also been transfected with the adenoviral E4ORF1 gene to overcome the hurdle of rapid cell death while starving primary endothelial cells [175]. Tumor endothelial cells can be isolated [171], and normal endothelial cells can be activated by using a co-culture with cancer cells or a cancer cell conditioned medium, as described [171].

#### 3.6.5. Endothelial Cell Culture Conditions

A variety of culture conditions for endothelial cells from various sources have been published. Primary endothelial cells are generally used at 2–8 passages to permit growth to confluence without undergoing senescence [177,187,188,192,198]. Culture in 30% FBS delays senescence until 18 passages [197]. ECFCs have been cultured to passages 7–10 [183].

Endothelial cell lines are generally cultured in endothelial growth medium-2 (EGM-2) with FBS concentrations of 10%, growth factors IGF-1, EGF, FGF-2 and additives, including hydrocortisone, ascorbic acid, heparin, GA-1000, glutamine and antibiotics [177]. When culturing on collagen-coated plates, lower concentrations of FBS of 5% were used [180].

Primary HUVECs and microvascular cells have been cultured in EGM-2, MEMα, M199 and MCDB mediums, 10–20% FBS and the additives endothelial growth supplement, glutamine and antibiotics on 0.2% gelatin-coated flasks with or without hydrocortisone and EGF [175,176,188,198]. Primary lung endothelial cells have been cultured in M199 medium, 15% FBS, EGF, heparin and glutamine [182]. Primary human aortic endothelial cells were cultured in EGM-2 with 8% FBS and antibiotics on gelatin-coated polystyrene plates [192]. Primary breast endothelial cells have been cultured in EGM-2 with 30% FBS, heparin, FGF-2, EGF, VEGF, long arginine-3 IGFR-1, ascorbic acid and hydrocortisone on collagen coated flasks [197]. FBS concentrations can be reduced to 5% after two passages [184] and kept at 2% for short-term 1–4 passages. Primary endothelial cells can be cryopreserved in 55% FBS and remain highly viable upon thawing [197]. Bone marrow endothelial cells can be isolated and expanded on fibronectin-coated plates in EGM-2, 5–10% FBS and endothelial growth medium [175,188]. Primary ECFCs have been cultured in EGM-2, RPMI-1640 and MEMα, 8–15% FBS and antibiotics without hydrocortisone on gelatin-coated plates [183,192,198]. Cells achieving quiescence can be maintained in RPMI 1640 supplemented with a low (2%) serum [171]. Primary endothelial cells transfected with the adenoviral E4ORF1 gene, called E4-EC, were cultivated in an M199 medium, supplemented with 20% FBS, βEGF and heparin in 2D and in DMEM-F12, 2% B27 supplement, insulin, FGF-2 and EGF and 0.2% methylcellulose to prevent cell aggregates [186].

HUVECs have been co-cultured with cancer cells in an EGM-2 medium with 1% FBS, growth factors and additives [179], but HUVECs used for co-culture experiments can be cultured as well without the addition of extra growth factors [198].

#### 3.6.6. Two-Dimensional Dormancy Cell–Cell Contact Co-Cultures

Several studies of endothelial cells and cancer cells in direct contact, mostly in 3D co-cultures, have been reported. In 2D co-cultures, human umbilical vein endothelial cells transfected with an adenovirus E4 open reading frame 1 vector co-cultivated with a commercially available bone marrow MSC line decreased the growth of sparsely seeded ER/PR- human-derived T4-2 cells [199] five-fold and induced quiescence in a laminin overlay organoid structure [12]. This was in contrast to co-cultures with MSC fibroblasts alone, where T4-2 cells grew freely [12]. Cell–cell contact during a 2D co-culture was required for the endothelial cell promotion of tumor growth.

Switching culture conditions between 2D and 3D collagen matrix-embedding induces plasticity in phenotype changes with a transient change to a progenitor cell-like phenotype and gene expression profile in 3D, a reversion upon returning to culturing in 2D and a reacquisition over time upon returning to 3D culturing [192]. These data suggest that a co-culture of endothelial cells with bone marrow stroma in 2D may likely modify their gene expressions and phenotypic characteristics and endow them with altered effects on cancer cells co-cultivated at a clonogenic density with the mixed cultures.

The effects of endothelial cells in the bone marrow microenvironment on cancer dormancy and reawakening in a stromal co-culture can be determined by generating bone marrow endothelial cells [175,188] from either murine or human bone marrow independently of and in parallel with bone marrow stromal fibroblast monolayer cultures, as described above. For convenience, the same bone marrow source can be used within an experiment. After reaching confluence in independent T25 flasks, bone marrow fibroblasts and bone marrow endothelial cells can be dispersed by trypsin treatment and mixed at variable ratios from 1:0, 10:1 and additional ratios all the way to 1:10 and 0:1, and cultured in αMEM/10% FBS with 2 mM of glutamine and antibiotics in quadruplicate 24-well tissue culture plates until confluent. Other culture media may also be used, depending on the experimental conditions. The concentration of endothelial cells at confluence should be assessed in one of the four wells at each ratio using labeled anti-CD31 or von Willebrand factor antibody [171], as the rates of growth of initial cell mixtures will vary by the consensus medium selected, and in co-culture in tissue culture-coated dishes. The confluent monolayers can be seeded with labeled cancer cells at a clonogenic density of 1000 cells/well and cultured in the same medium for 7–9 days, along with any intended molecular perturbations needed to test a particular hypothesis.

Because of the potentially diverse molecular, cellular and phenotypic conclusions of co-culture experiments on cancer cell behavior, it is imperative to characterize and standardize all of the co-culture variables. In addition to the monolayer cell sources, mixture ratios, culture conditions, media, additives, consensus media, levels of stroma/endothelial cell confluence and proliferative status of the endothelial cells at the time of the seeding of cancer cells, the cancer-cell-related variables will have to be determined as well. The cycle status of endothelial cells should be determined and can be accomplished by immunofluorescence Ki67 antibody staining.

It is important to control and isolate the co-cultivated endothelial cells and stromal fibroblasts before and after co-cultivation with cancer cells to determine the mutual effects on gene-expression profiles that the different cells have on each other as part of an initial comprehensive characterization of the experimental model. Cancer cells are seeded at clonogenic density, but if they proliferate, they can induce changes on stroma and endothelial cells during the co-cultivation that affect the stroma’s influence on the cancer cells. This effect depends on the concentration of the cancer cells, which may reach a critical mass and result in a feedback signal loop, and on the time length of exposure. The medium and additives should be the minimum that permit cancer cell proliferation, permit cancer cell dormancy and permit stromal and endothelial cell quiescence at the time the dormancy is induced in the cancer cells. The factors being investigated for their ability to induce cancer cell dormancy should be considered carefully so as not to affect endothelial cell activity as well. Gene disruptions or enforced gene expressions in cancer cells to test hypotheses or their effects on dormancy and reawakening can be completed before a co-incubation. However, antibodies or small interfering RNAs may have to be added on successive days after the start of a co-culture, and this may affect the cells in the substratum as well. Clearly, all variables must be tested on the system and rigorously controlled before conclusions from derived data can be sustained. While the 2D modular stromal approach to studying dormancy is a powerful system for revealing the mechanisms and effects on cancer cells by stromal cells and components, it must be wielded with exceptional caution and controlled for all potential variables in order to generate hypotheses to be tested in vivo.

### 3.7. Other Components

There are additional components of the bone marrow microenvironment that can be incorporated in a co-culture system. These include cellular components that are immunomodulatory, such as macrophages, [152,173,200,201,202], T-cells [101] and NK cells [203,204], for example, structural components, such as heparan sulfate proteoglycans [87,205,206], fibronectin [79,89], tenascin, versican, biglycan, periostin and TGFβ [12], as well as a wide range of growth factors and cytokines having roles in the niche. The addition of structural components to the media, such as fibronectin, may not necessarily replicate biological conditions in vivo that rely on their structural organizations for pro-dormancy effects [89] and may yield proliferative effects [92,207]. Hypoxic conditions can be generated in a co-culture to more accurately model the relevant biologic conditions that affect dormancy in vivo [177,208,209,210], and temperature can be modulated to modify adipogenic differentiation [70]. The individual design would have to be constructed with step-by-step developments, testing, evaluation and validation of the many variables involved in such a multi-component system.

## 4. Conclusions

There are several general concepts addressed in this manuscript. The bone marrow microenvironment is complex and can be modeled in vitro with variable components to determine their effects on cancer cell growth or dormancy and on each other. Cellular components can be generated independently and mixed with stromal fibroblasts at different ratios. They will grow at different rates after mixing, making it imperative to determine the ratios of different cells in the final monolayer in order to correlate their effects on seeded cancer cells. Bone marrow niche-residing cells also differ in their potential to differentiate after mixing in a culture. Therefore, the medium and additives used during the time these cells grow to confluence must be chosen carefully. It is important to use the simplest medium necessary for cancer cell growth for co-cultivation once the monolayer components have been differentiated, lest they continue to differentiate during the week to 10 days of co-culture with seeded cancer cells.

Cancer cells must be seeded at clonogenic density to ensure that their interactions are solely with the substratum and not with each other. Moreover, cancer cells can modify stroma and confer novel traits that have the ability to feedback promote cancer growth. This is minimized at low cancer cell/stroma ratios achieved at a clonogenic density of cancer cells and by limiting the assay to less than 10 days. However, various stromal cells can affect each other’s behavior and differentiation as well, and different ratios may result in different mutual effects in the mixture during the growth to confluence. Because of these effects, the system can be used to study niche components as an investigational tool.

It is important to characterize the system components by immunophenotyping the culture at confluence and determining the cells’ cycle status. This will generate a full understanding of these dynamics before using the system to determine its effects on cancer cells and generating conclusions on the biology of interactions. It may also be necessary in some scenarios to characterize the immunophenotype and differentiated status of stroma cells after co-cultured cancer cell growth or dormancy is assessed to fully understand the status of the stroma cells at the end of the cancer co-culture. These control characterization studies of complex cell combinations are essential to ensure the understanding of the status and representation of the cells generating the data used for biological interpretations.

Additional cellular components can be added to confluent fibroblast cultures or mixed cultures, including T-lymphocytes, NK cells and macrophages of specific immunophenotypes. These can be added together with cancer cells to the monolayers to determine their effects on cancer proliferation, survival and dormancy. It is advisable to characterize the culture at the end of the experiment. Similarly, structural and soluble components of the microenviroment can be added to test their effects on cancer cells.

## Data Availability

Data available upon request.
